# The use of array-CGH in a cohort of Greek children with developmental delay

**DOI:** 10.1186/1755-8166-3-22

**Published:** 2010-11-09

**Authors:** Emmanouil Manolakos, Annalisa Vetro, Konstantinos Kefalas, Stamatia-Maria Rapti, Eirini Louizou, Antonios Garas, George Kitsos, Lefteris Vasileiadis, Panagiota Tsoplou, Makarios Eleftheriades, Panagiotis Peitsidis, Sandro Orru, Thomas Liehr, Michael B Petersen, Loretta Thomaidis

**Affiliations:** 1 Bioiatriki S.A., Laboratory of Genetics, Athens, Greece; 2 Dipartimento di Patologia Umana ed Ereditaria, Universita di Pavia, Pavia, Italy; 3 Department of Obstetrics and Gynecology, University of Thessaly, Larissa, Greece; 4 Department of Ophthalmology, University of Ioannina, Ioannina, Greece; 5 Department of Neurology, 251 General Hellenic Air Force Hospital, Athens, Greece; 6 EmbryoCare, Fetal Medicine Centre, Athens, Greece; 7 Department of Fetal Medicine, ''Royal Free Hospital" London, UK; 8 Department of Medical Genetics, University of Cagliari, Binaghi Hospital, Cagliari, Italy; 9 Jena University Hospital, Institute of Human Genetics and Anthropology, Jena, Germany; 10 Department of Genetics, Institute of Child Health, Athens, Greece; 11 Department of Pediatrics, University of Athens, ''Aglaia Kyriakou'' Children's Hospital, Athens, Greece

## Abstract

**Background:**

The genetic diagnosis of mental retardation (MR) is difficult to establish and at present many cases remain undiagnosed and unexplained. Standard karyotyping has been used as one of the routine techniques for the last decades. The implementation of Array Comparative Genomic Hybridization (array-CGH) has enabled the analysis of copy number variants (CNVs) with high resolution. Major cohort studies attribute 11% of patients with unexplained mental retardation to clinically significant CNVs. Here we report the use of array-CGH for the first time in a Greek cohort. A total of 82 children of Greek origin with mean age 4.9 years were analysed in the present study. Patients with visible cytogenetic abnormalities ascertained by standard karyotyping as well as those with subtelomeric abnormalities determined by Multiplex Ligation-dependent Probe Amplification (MLPA) or subtelomeric FISH had been excluded.

**Results:**

Fourteen CNVs were detected in the studied patients. In nine patients (11%) the chromosomal aberrations were inherited from one of the parents. One patients showed two duplications, a 550 kb duplication in 3p14.1 inherited from the father and a ~1.1 Mb duplication in (22)(q13.1q13.2) inherited from the mother. Although both parents were phenotypically normal, it cannot be excluded that the dual duplication is causative for the patient's clinical profile including dysmorphic features and severe developmental delay. Furthermore, three *de novo *clinically significant CNVs were detected (3.7%). There was a ~6 Mb triplication of 18q21.1 in a girl 5 years of age with moderate MR and mild dysmorphic features and a ~4.8 Mb duplication at (10)(q11.1q11.21) in a 2 years old boy with severe MR, multiple congenital anomalies, severe central hypotonia, and ataxia. Finally, in a 3 year-old girl with microcephaly and severe hypotonia a deletion in (2)(q31.2q31.3) of about ~3.9 Mb was discovered. All CNVs were confirmed by Fluorescence *in situ *hybridization (FISH). For the remaining 9 patients the detected CNVs (inherited duplications or deletions of 80 kb to 800 kb in size) were probably not associated with the clinical findings.

**Conclusions:**

Genomic microarrays have within the recent years proven to be a highly useful tool in the investigation of unexplained MR. The cohorts reported so far agree on an around 11% diagnostic yield of clinically significant CNVs in patients with unexplained MR. Various publicly available databases have been created for the interpretation of identified CNVs and parents are analyzed in case a rare CNV is identified in the child. We have conducted a study of Greek patients with unexplained MR and confirmed the high diagnostic value of the previous studies. It is important that the technique becomes available also in less developed countries when the cost of consumables will be reduced.

## Background

Mental retardation (MR) is a common disorder for which the genetic diagnosis in many instances is lacking. The detection rate of chromosomal abnormalities in patients with MR and dysmorphic features has increased due to the improvements of molecular cytogenetic methods. Standard cytogenetic methods cannot detect imbalances smaller than 5-10 Mb and the detection rate of visible chromosomal abnormalities in patients with moderate to severe MR is 3.7% [1]. The development of Fluorescence *in situ *hybridization (FISH) probes for the subtelomeric regions has led to the identification of cryptic unbalanced rearrangements in an additional 2.5-3% of patients with moderate/severe MR of unknown cause [2-4].

Molecular karyotyping (MK) through array-CGH or Single Nucleotide Polymorphisms array (SNP-array) is rapidly becoming the first tier clinical genetic test for patients with unexplained developmental delay/intellectual disability, autism spectrum disorders, and multiple congenital anomalies. Numerous studies have clearly demonstrated that MK offers a much higher diagnostic yield (15%-20%) for this group of patients in respect to conventional karyotyping with a G-banded karyotype (approximately 3%, excluding Down syndrome and other recognizable chromosomal syndromes), primarily because of its higher sensitivity for submicroscopic deletions and duplications [5].

The widespread application of this technique has lead to the identification of large-scale copy number polymorphisms (CNPs), shown to contribute substantially to genomic variation [6,7], and segmental duplications were found to define hotspots of chromosomal rearrangements [8].

Here we report the first Greek experience from a cohort of 82 children with learning disabilities and dysmorphism, in whom subtelomeric chromosomal abnormalities were excluded by FISH or MLPA techniques. All children presented with various degrees of unexplained MR or learning difficulties (MR/LD) and facial dysmorphism/congenital malformations, suggestive of chromosomal anomalies: a) not associated with congenital brain malformation (CBM) in either CT or MRI brain scan, b) associated with CBM in either CT or MRI brain scan without improvement and c) associated with CBM in either CT or MRI brain scan with improvement.

## Results

Eighty-two patients with unexplained MR and presence of features suggestive of a chromosomal anomaly were analyzed by array-CGH. All patients had an apparently normal karyotype when investigated by standard GTG-banding (> 550 band resolution per haploid karyotype). In addition, subtelomeric FISH and MLPA analyses were performed without revealing any rearrangements. Among the 82 patients analyzed, 13 (15.8%) were found to have cryptic chromosomal imbalances: 6 patients with duplications, 5 patients with deletions, one with triplication and one patient with two duplications. Array-CGH results and phenotype of these individuals are given in Table 1. For all 13 patients, array-CGH analysis has been extended to parental samples, so to establish if an aberration was inherited or de novo. In 3 out of the 13 patients the chromosomal rearrangements occurred *de novo*; these aberrations were classified as causative for the phenotype. The other 11 aberrations which were observed in the remaining of 10 patients, were considered as likely benign since they have not previously been reported and have been inherited from a healthy parent. In total, 3 *de novo *clinically significant CNVs were identified in 3 out of the 82 patients (3.6%) and 11 abnormalities with uncertain clinical significance were detected in 10 patients (12.2%). All three patients presented with CBM. Two patients were of subgroup c (Background) associated with CBM and improvement and one patient was of subgroup b associated with CBM and no improvement after intervention. The median de Vries score] of all 82 patients was 4.47 (range 0-9).

**Table 1:** Clinical information and array-CGH results in Greek children with unexplained mental retardation

Case/no	Gender	MR	Array-CGH results	Origin	Estimated size	Clinical Features
1	F	Moderate	tripl(18)(q21.1) 42,812 Mb-48,558 Mb	de novo	6 Mb	maxillary hypoplasia, small jaw, prominent occiput, hypertelorism, epicanthal folds, downward slanting palpebral fissures with sunken eyes and long eyelashes. Brain MRI scan suggested periventricular leukomalacia (PVL).
2	M	Severe	dup(10)(q11.1q11.21) 46,568 Mb-51,264 Mb	de novo	6 Mb	corpus callosum hypoplasia, mild dilatation of subarachnoid areas and frontotemporal atrophy, severe central hypotonia, ataxia, triangular face, enlarged cranium cerebrale, bifid scrotum, cryptorchidism, ulnar deviation of both elbows, deep palmar creases of hands and feet and syndactyly of 2nd and 3rd toes bilaterally.
3	F	Moderate	del(2)(q31.2q31.3) 178,393 Mb-182,296 Mb	de novo	3,9 Mb	severe hypotonia with microcephaly, Brain MRI scan showing dilated lateral ventricles and diminishing white matter at the level of the trigons bilaterally. Speech limited to simple vocalization with lack of meaning. Her overall developmental level was equivalent to 8 months, which corresponds to a developmental quotient (D.Q.) = 30.
4	M	Severe	del(6)(p21.2) 38,420 Mb-38,554 Mb	paternal	135 kb	mild dysmorphic facial features (epicanthial folds, hypertelorism and auricle abnormalities)normal growth parameters severe mental retardation DQ 45, behavioural disorders with autistic features. motor disorder, hypotonia of central origin, brain malformation,(pituitary cyst), no cognitive improvement after intervention
5	F	Moderate	dup(15)(q13.3) 29,809 Mb-30,298 Mb	maternal	0.5 Mb	mild dysmorphic facial and body features, such as hrinolalia with high-pitched voice, epicanthus, myopia, clinodactyly, and wide internipple distance
6	F	Moderate	dup(20)(p11.21) 25,375 Mb-25,420 Mb	paternal	80 kb	microcephaly, cleft palate, somatometric parameters below the 3^rd ^centile, perimembranous ventricular septal defect, hyperopia, prominent forehead, synorphrys, long eyelashes, bulbous nasal tip, smooth philtrum, thin upper lip, hirsutism, and bilateral clinodactyly of the 5^th ^finger
7	M	Moderate	dup(16)(q22.1) 68,390 Mb-68,534 Mb	maternal	150 kb	dysmorphic facial features, motor disorder, epicanthus, hyperopia
8	M	Moderate	dup(16)(p11.2) 27,741 Mb -27,919 Mb	paternal	200 kb	mild microcephaly and dysmorphic facial features, maxillary hypoplasia, epicanthal folds, up-slanting palpebral fissures, long eyelashes and hypertelorism, auricle abnormalities and his mouth characterized by a long philtrum with gothic palate
9	F	Severe	del(1q)(31.3) 196,153 Mb-196,532 Mb	maternal	400 kb	somatometric parameters below the 3^rd ^centile, mild dysmorphic facial features, auricle abnormalities, developmental and motor delay
10	M	Severe	del(11)(q21) 94,602, Mb-95,086, Mb	paternal	500 kb	multiple gestation (triplex-IVF), dysmorphic facial features, squint, flat filtrum, frontal bossing, epicanthus, auricle abnormalities, macrocephaly, fronto-temporal brain atrophy, motor disorder, hypotonia of central origin, severe mental delay, severe behavioral disorders
11	M	Severe	dup(17)(q25.1) 70,793 Mb-71,106 Mb	paternal	300 kb	neonatal hypotonia, macrocephaly, dolicocephaly, mild dilation of subarachnoid area, severe developmental and motor delay, cryptorchidism, mild dysmorphic, facial features, epicanthus, clinodactyly, short neck, large distance between nipples, short vraxionas
12	M	Moderate	del(1)(p12) 118,688 Mb-119,490 Mb	paternal	800 Kb	mild dysmorphic facial features, bulbous nose, short fingers, clinodactyly, upward slanting palpebral fissures, moderate learning difficulties, language delay with phonological problems and stuttering
13	M	Severe	dup(3)(p14.1) 65,912 Mb-66,462 Mb	paternal	550 kb	severe motor disorder (marked spastic tetraplegia), dysmorphic facial features (prominent forehead, low-set ears, epicanthal folds, flat philtrum, and long eyelashes), severe developmental delay as his developmental level was equivalent to 5 months.
			dup(22)(q13.1q13.2) 38,255 Mb-39,383 Mb	maternal	1.1 Mb	

## Discussion

Array-CGH has proven to be an important tool to detect submicroscopic chromosomal aberrations. We used DNA oligonucleotides to study 82 patients with normal karyotype in whom a chromosomal abnormality was suspected due to the combination of clinical features. All 82 patients presented with MR or LD, dysmorphic facial features and congenital malformations. In order to better define the clinical features correlated with chromosomal imbalance, we divided the cohort in three subgroups.

a) 24/82 (29.3%) patients with MR/LD, facial dysmorphism, congenital malformations not associated with CBM,

b) 40/82 (48.8%) patients with MR/LD, facial dysmorphism, congenital malformations associated with CBM and no improvement of cognitive skills after intervention,

c)18/82 (21.9%) patients with MR/LD, facial dysmorphism, congenital malformations associated with CBM and improvement of cognitive skills after intervention.

All patients showed a normal G-banded karyotype, and in all of the cases telomere rearrangements had been excluded by FISH or MLPA.

**Figure 1:**
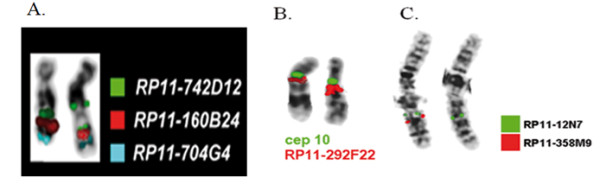
**FISH analysis for the confirmation of a-CGH results in the three de novo imbalances**. **A**. In patient 1 the analysis performed using BAC probes RP11-742D12 in 18q21.1 (triplicated), RP11-160B24 in 18q21.2 (normal) and RP11-704G4 in 18q23 (normal). **B**. In patient 2 it was used BAC probe RP11-292F22 in 10q11.22 (duplicated) and cep10 probe (normal) **C**. Lastly in patient 3 BAC probes RP11-12N7 in 2q31.1 (normal) and RP11-358M9 in 2q31.3 (deleted) were applied.

In the 82 patients, 14 chromosomal imbalances were detected (~17%). Three of the observed chromosomal aberrations were de novo and eleven aberrations were inherited from one of the phenotypically normal parents. The array-CGH results were confirmed by FISH technique in all three *de novo *cases (Figure 1).

The *de novo *triplication (**Patient 1**) in 18q is reported here for the first time. It involves chromosome band 18q21.1 and it spans a region of about 5.8 Mb. It is known that Edwards syndrome is mostly associated with trisomy 18, however several individuals with partial trisomy of the long arm of chromosome 18 have been reported. In these cases patients display manifestations ranging from a relatively mild to a severe phenotype. Genotype-phenotype correlations have suggested that duplication of regions (18)(q12.1q21.2) is critical for the trisomy 18 phenotype [9,10], while the relationship between duplication of the other 18q regions and mental retardation, growth delay, and dysmorphism is less clear. Our patient displayed only mild dysmorphic features and speech delay. Only one other case with duplication in this area but in a much larger region has been reported [11]. It concerns a 9 year-old boy with profound MR and growth delay. This individual was diagnosed with a duplication involving (18)(q12.3q21.31), which spans a region much wider than the one reported here. Neither the boy nor the girl in our study display the typical Edwards syndrome phenotype but both individuals share some clinical features like failure to thrive, slanting palpebral fissures and ventricular septal defect. The absence of seizures in our case and the difference in severity of MR and growth delay are probably related to the difference in the size of the duplicated region. It is possible that the mild phenotype in our case is due to the fact that the region does not include genes influencing physical development, or that the triplication does not alter significantly the expression pattern of the corresponding genes.

**Figure 2:**
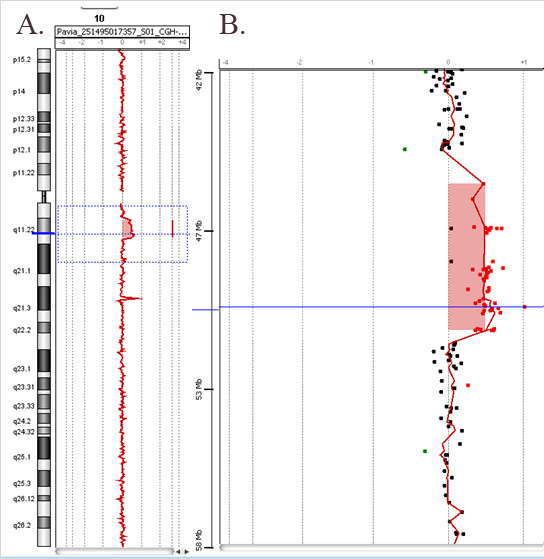
**Array-CGH profile of chromosome 10 showing an interstitial duplication (patient 2)**. **A**. The whole chromosome 10 view. **B**. The enlarged view of the rearrangement as provided by Agilent Technologies, CGH Analytics 3.5.14. The proximal duplication breakpoint was between 45,478 Mb and 46,578 Mb. The distal duplication breakpoint was between 51,264 Mb and 51,676 Mb. The overall size of the duplication was about 4.8 Mb.

Proximal 10q duplication (**Patient 2**) is a well defined but rare genetic syndrome [12-19]. This represents the first case of partial proximal trisomy 10q characterized by array-CGH (Figure 2). The typical profile of partial proximal trisomy 10q syndrome includes postnatal growth retardation, microcephaly, and mild to moderate developmental delay. Frequent dysmorphic features are prominent forehead, small deep-set eyes, epicanthus, upturned nose, bow-shaped mouth, micrognathia, flat and thick ear helices, and long slender limbs. In concordance, our patient showed severe central hypotonia, ataxia, triangular face, enlarged cranium cerebrale, bifid scrotum, cryptorchidism, ulnar deviation of both elbows, deep palmar creases of hands and feet, and syndactyly of 2nd and 3rd toes bilaterally. It is noteworthy that the pericentromeric region 10p11.2 to 10q11.2 has been reported to contain unbalanced chromosomal abnormalities without phenotypic consequences [20].

**Figure 3:**
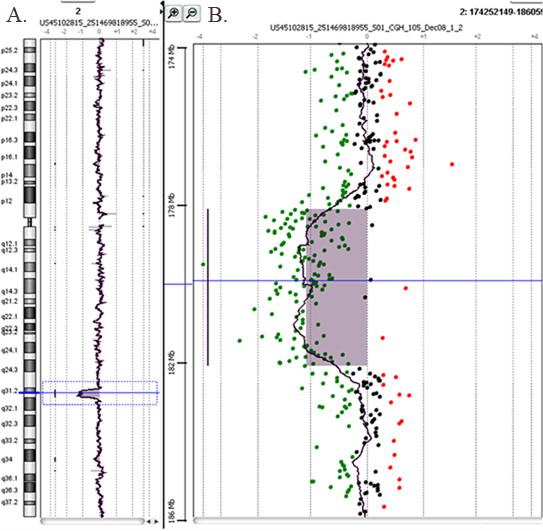
**Array-CGH profile of chromosome 2 showing an interstitial deletion (average log2 ratio: -0.83) (patient 3)**. **A**. The whole chromosome 2 view. **B**. The enlarged view of the rearrangement as provided by Agilent Technologies, CGH Analytics 3.5.14. The proximal deletion breakpoint was between 178,370 Mb and 178,393 Mb, and the distal deletion breakpoint was between 182,296 Mb and 182,369 Mb. The overall size of the deletion was about 3.9 Mb.

Deletions involving 2q31-q32 (**Patient 3**) (Figure 3) have been reported in more than 30 patients [21-26]. Most mutations, involving the 2q31 segment, comprised the HOXD gene cluster which plays an important role in limb development. In our case, the patient showed no limb malformation as the deletion did not involve the HOXD gene cluster.

Dual chromosomal duplication **(Patient 13) **is a rarely reported genetic condition. To our knowledge this is the first case of simultaneous duplication involving 3p14.1 and 22q13.1-q13.2. Pramparo *et al*. (2008) [27] identified for the first time a 7 Mb duplication of (22)(q13.1q13.2) in a 10-year-old girl with dysmorphic features (prominent forehead, small low-set ears, hypertelorism, epicanthal folds, small palpebral fissures, short philtrum, and syndactyly), brain-MRI clinical findings (signal abnormalities in the periventricular white matter, hypoplastic corpus callosum, under-rotated hippocampus on the left and atrophic hippocampus on the right side), moderate MR, and severely disorganized mood and behavior with periodic manic episodes. The authors showed that the duplication was the result of a maternal intrachromosomal insertion. The 7 Mb duplicated region is gene-rich, carrying > 120 known genes and ~50 novel transcripts, presumably including genes whose copy number increase is most likely associated with the phenotypic features. Our patient's chromosome 22 duplication is smaller in size (~1.1 Mb) but still contains 9 genes of known function (8 fully-sized and one partial gene sequence at the proximal breakpoint). The clinical features of our patient have common characteristics with those of the patient published by Pramparo *et al*. (2008), mainly with regard to malformations. We anticipate that the ~1.1 Mb duplicated chromosome 22 region is associated with the patient's phenotype, while some contribution from the 550 kb 3p14.1 duplication should also be considered. The latter assumption is supported by the fact that the chromosome 22 duplication alone was detected in the phenotypically normal mother. It cannot be excluded that the dual duplication in this patient is causative for the patient's phenotype, as also suggested by the double hit model proposed recently, saying that two events (two CNVs) act in concert to produce a more severe phenotype [28].

Nine other patients (**Patients 4-12**) with developmental delay and various clinical features were found to have deletions or duplications inherited from one of the parents (Table 1). The size of these chromosomal aberrations ranged between 80 kb and 500 kb. However, none of those appears to be associated with the patients' phenotype since they were inherited from a healthy parent. Therefore, these CNVs can be classified as likely benign [29,30] and the underlying causes for the abnormal phenotypes remain unknown.

## Methods

### Patients

A total of 82 Greek children were referred to the Department of Pediatrics, ''Aglaia Kyriakou'' Children's Hospital, Athens for developmental assessment from 2007 to 2009. All patients were scored using a clinical scoring system [8]. Ages varied between one and thirteen years, with a mean age of 4.9 years. A total of 46 boys and 36 girls were analyzed. All patients had normal karyotype (G-banding analysis at resolution of 550 bands per haploid karyotype, ISCN 2005) and subtelomeric abnormalities determined by MLPA or FISH techniques had been excluded. Written informed consent was obtained from the parents of all patients.

### DNA isolation

Blood samples with EDTA were collected from patients and parents. DNA extraction was carried out using the Qiagen QIAamp^® ^DNA blood mini kit (QIAGEN, Valencia, CA, USA).

### Array-CGH

Array-CGH analysis was performed using 4 × 44 K, 2 × 105 K and 4 × 180 K commercial arrays (Agilent Technologies, Santa Clara, CA, USA) according to the manufacturer's instructions. This platform contains 60-mer oligonucleotide probes spanning the entire human genome with an overall median probe spacing of 22 Kb (19 Kb in Refseq genes). A sex-matched reference DNA (NA10851, NA15510, Coriell Cell Repositories) has been used for each subject tested. Previously reported benign CNVs listed in the Database of Genomic Variants http://projects.tcag.ca/variation/ were excluded from further analysis. After hybridization, the arrays were scanned in a dual-laser scanner (DNA Microarray Scanner with Sure Scan High-Resolution Technology, Model G2565CA, Agilent Technologies) and the images were extracted and analyzed through Agilent Feature Extraction software (v10.5.1.1) and DNA Analytics software (v4.0.73), respectively. Changes in test DNA copy number at a specific locus are observed as the deviation of the log_2_ratio value from the value of 0 of at least three consecutive probes. The quality of each experiment was assessed by using a parameter provided by Agilent software (QC metric) and on the basis of DNA quality.

### Bioinformatics

Copy number changes identified in the samples were compared to the Database of Genomic Variants http://projects.tcag.ca/variation/ and also visualized by using the UCSC Genome Browser website http://genome.ucsc.edu/. The positions of oligomers refer to the Human Genome March 2006 assembly (hg18).

### FISH analysis

All three significant de novo aberrations were confirmed by metaphase FISH using BAC clones (RP11-742D12, RP11-160B24 and RP11-704G4 for patient 1, RP11-292F22, RP11-463P17 and RP11-164N7 for patient 2 and RP11-12N7 and RP11-358M9 for patient 3) in the same region as the deletions or duplications identified by microarray analysis [31]. Metaphase chromosomes were obtained from blood lymphocytes according to a standard protocol [32].

### MLPA analysis

MLPA analysis was carried out using P036 and P070 probes purchased commercially from MRC-Holland (Amsterdam, Netherlands). The procedure was carried out according to the manufacturer's protocol. Briefly, 100 ng DNA was denatured at 98°C and hybridized overnight at 60°C with the SALSA probe mix P036 and P070. The next day, samples were treated with Ligase 65 for 15 min at 54°C. The reaction was stopped by incubating the samples at 98°C for 5 min. Finally, the amplification step was carried out using the SALSA PCR FAM-labeled primers. The amplification products were analyzed on an ABI 3130 Genetic Analyzer (Applied Biosystems, Carlsbad, CA, USA) using 36 cm capillaries and POP-7 polymer. The obtained data were analyzed using Genemapper 4 Software. The final analysis of the MLPA data was carried out using the Coffalyser Software.

## Competing interests

The authors declare that they have no competing interests.

## Authors' contributions

EM wrote the manuscript; AG, LV, PP and LT referred the patients for study; LT coordinated the clinical analysis of the patients; EM performed the cytogenetic analysis; AV, KK, SR, and EL signed out the array-CGH analysis results; SO and PT were responsible for the MLPA analysis; GK performed the ophthalmologic examination; TL was responsible for the FISH analyses; MBP, ME and EM coordinated the study. All authors have read and approved the manuscript.
